# Frozen Fruit and Vegetable Perceptions and Usage among a Multistate Sample of Supplemental Nutrition Assistance Program Education and Expanded Food and Nutrition Education Program Participants

**DOI:** 10.1016/j.cdnut.2026.107640

**Published:** 2026-01-17

**Authors:** Annie J Roe, Gemma E Bastian, Raveen Rani, Joslyn K Russell

**Affiliations:** 1 Margaret Ritchie School of Family and Consumer Sciences, University of Idaho, Moscow, ID, United States; 2 School of Health and Human Sciences, South Dakota State University, Brookings, SD, United States; 3 Department of Biology and Microbiology, South Dakota State University, Brookings, SD, United States; 4 Department of Health, Nutrition, and Food Sciences, Florida State University, Tallahassee, FL, United States

**Keywords:** frozen foods, food preferences, low socioeconomic status, frozen fruits and vegetables, Supplemental Nutrition Assistance Program—Education, Expanded Food and Nutrition Education Program

## Abstract

**Background:**

Despite the health benefits of a diet rich in fruits and vegetables (FV), most Americans do not consume the recommended amounts. Federal nutrition education programs for adults with low income promote frozen fruits and vegetables (FFV) to improve dietary intake, save money, and prevent food waste. However, FFV purchasing, use, and attitudes among this consumer base are not well understood.

**Objectives:**

The aim of this study is to determine the perceptions and use of FFV among adults who have participated in Supplemental Nutrition Assistance Program—Education (SNAP-Ed) or Expanded Food and Nutrition Education Program (EFNEP) classes.

**Methods:**

An electronic survey was developed to assess frozen food purchases, FFV perceptions and preparation, and nutrition education and outreach. Subjects were recruited via SNAP-Ed/EFNEP programs. Descriptive analyses were conducted using SAS 9.4 (SAS Institute Inc., Cary, NC, USA).

**Results:**

Subjects (*n* = 421) were mostly female (84%), without a college degree (72%), and with annual incomes <$25,000 (63%). Roughly one-third of respondents reported eating frozen foods every few days or almost every day, one-third ate them weekly, and one-third ate frozen foods a few times per month or year. Most (59%) considered using SNAP benefits to buy frozen foods very or extremely important. The top reasons for purchasing FFV were long shelf life (38%), ease of preparation (36%), and convenience (34%). When comparing FFV to fresh FV, participants considered FFV to result in less food waste but considered fresh FV to have fewer preservatives, better appearance, texture, nutrition, and flavor.

**Conclusions:**

SNAP-Ed/EFNEP-participating adults are a key consumer demographic for frozen foods, using them to save money and limit food waste. However, misconceptions persist, such as that fresh FV are more nutritious than frozen. Nutrition education programs for adults with low income could incorporate additional content on adding FFV into a healthy diet to help participants use these cost-effective foods to meet their nutrition and health goals.

## Introduction

The consumption of fruits and vegetables (FV) is crucial for maintaining overall health and lowering the risk of chronic conditions like obesity, heart disease, type 2 diabetes, and certain cancers [1]. However, in 2019, only 12.3% of Americans consumed enough fruits, and only 10% consumed enough vegetables to meet the recommended daily intake [2]. Frozen fruits and vegetables (FFV) offer a promising solution to fill this nutritional gap, particularly for families with limited income. Although FFV are not always less expensive than fresh varieties, their longer shelf life can reduce food waste and save consumers money over time [3]. These savings from reduced food waste may be especially advantageous for households with limited food budgets, where the cost of spoiled fresh FV can be a significant barrier to meeting FV intake recommendations [4].

Studies have shown that FFV have nutrition profiles comparable to fresh FV, and that individuals consuming frozen produce tend to consume more FV overall, have higher potassium intake, and reduced sodium consumption [5]. Despite these benefits, consumer perceptions are still complicated, with persistent concerns about taste and nutritional content influencing purchasing decisions [[6], [7], [8]].

Nutrition education programs for audiences with low income, namely the Expanded Food and Nutrition Education Program (EFNEP) and Supplemental Nutrition Assistance Program Education (SNAP-Ed), are potential avenues by which to influence consumer perception and behavior around frozen foods, particularly FFV [9]. Through hands-on classes using evidence-based curricula, peer-educators and nutrition professionals work with community partners to promote lasting behavior changes related to diet quality, physical activity, food safety, and food resource management [10,11]. These interventions have been found to be effective in changing dietary behaviors, with research showing that interventions in meal planning, price comparison, and food budgeting are most likely to improve diet quality [12]. Nutrition education plays a crucial role in shaping food choices, particularly among populations with low income who may have limited access to accurate nutrition information [13]. Studies document that individuals with high nutrition literacy make more informed choices of foods and have improved diet quality, leading to better health outcomes [13]. Behavioral nutrition education through EFNEP and SNAP-Ed could help eliminate myths about frozen foods, emphasizing their diverse options, affordability, nutritional value, and reduced food waste. The purpose of this study was to describe the perceptions, purchase drivers, and use of FFV among adults with low income who have participated in SNAP-Ed or EFNEP classes.

## Methods

### Study design and participants

This cross-sectional multistate study aimed to describe the perceptions and behaviors related to frozen food consumption, particularly FFV among EFNEP and SNAP-Ed participants. Self-reported data were collected via an online survey, administered through Qualtrics, and made available from November 2023 to September 2024 (Supplementary Material; Qualtrics Survey Questions).

Recruitment was facilitated via partnerships with SNAP-Ed and EFNEP programs at 15 land-grant universities representing each of the 4 geographic regions (Western, North Central, Southern, and Northeast) defined by the USDA, National Institute of Food and Agriculture (USDA NIFA). Researchers provided study flyers to each program, which were distributed to participants by SNAP-Ed and EFNEP educators. Individuals aged ≥18 y who reported consuming frozen food more often than “never,” had participated in ≥1 SNAP-Ed or EFNEP lesson, and could read English or Spanish were eligible.

To prevent unintended participants from taking the survey (e.g., family and friends of SNAP-Ed/EFNEP participants who were not attending lessons, or learning-language models trained to scour survey links online to collect payment), a state-specific password was given out verbally by the educators. The participant had to enter the password that corresponded with the state they lived in to continue with the study. On survey completion, all participants received a $20 electronic gift card. This study was approved by the Institutional Review Board at South Dakota State University (#IRB-2305001-EXM).

### Measures

Throughout the survey, frozen foods were defined as items found in the frozen aisles of the grocery store, including ice cream, pizzas, prepared meals or entrees, fries or other potato products, breakfast items, fruits, vegetables, and meat/seafood. Not included were bagged ice and items that individuals froze themselves. FFV were further defined as including individual vegetables, mixed vegetables, potato-based items, mixed fruit, individual fruit, prepared vegetables, spiralized/riced vegetables, smoothie mixes, and fruit and yogurt bites. Survey domains used by the researchers were based on prior market research conducted by the American Frozen Food Institute [14]. Most questions were either multiple choice or used 4-to-6-point rating scales.

#### Frozen food purchases and consumption

Participants were asked about the frequency of both their purchasing and consumption of frozen foods, including FFV. They also reported how important it was for them to use SNAP benefits to purchase frozen foods. Moreover, participants reported the top reasons for purchasing FFV and types of FFV typically purchased. Barriers associated with purchasing frozen food and conditions that would increase purchase of FFV were also captured.

#### FFV perceptions and use

Participants were asked to compare fresh and frozen produce in attributes such as nutrition and flavor. For each attribute, participants could choose fresh as being better, frozen as being better, or both as being equal. Further, participants were asked how they used FFV, and for what reasons they chose to use frozen produce over fresh. Participants reported on how often they were the primary food preparer in the household, frequency of FFV preparation, and in what types of dishes they used these foods in (e.g., appetizers, casseroles, or desserts).

#### Nutrition education and outreach

Participants were asked to select all from a list of nutrition education topics about frozen foods that they would be interested in learning about; they could also provide their own suggestions for educational topics relating to frozen foods. They also reported the likelihood that they would engage in certain educational outreach channels, such as at grocery stores or supermarkets, social media, or QR codes linking to informational websites. Participants were also asked if they ever met or consulted with a registered dietitian at a grocery store or supermarket, and how often they read nutrition facts labels on frozen foods.

#### Demographics

Participants reported their age, gender, race, and ethnicity, highest educational attainment, and annual household income. All demographic information was chosen by the participant from a provided list of options.

### Statistical analysis

Descriptive analyses were conducted using SAS 9.4. The data were cleaned to remove participants with incomplete responses or participants who were likely not recruited from SNAP-Ed or EFNEP classes (e.g., if a participant’s response was submitted from a geolocation in another country). If it seemed like the survey link was shared with family and friends (e.g., multiple participants with the same surname, or completing from the same IP address), one of the investigators followed up with the respondents via email to confirm which ones participated in SNAP-Ed or EFNEP classes. The data were analyzed for the full sample, but in some instances, descriptive statistics were stratified based on either the number of SNAP-Ed/EFNEP lessons the participant had completed when they filled out the survey (1–3, 4–6, 7–9, or ≥10) or their frequency of preparing and consuming frozen foods (core consumers reported consuming frozen foods daily or every few days; medium consumers reported doing so weekly or every other week; and low consumers reported doing so every few weeks or less than monthly). Chi-square was used to assess the frequency distribution of responses to fresh compared with frozen FV attributes and relationships between the number of nutrition education lessons and fresh compared with frozen FV attributes. Significance was set at *P* ≤ 0.05.

## Results

### Subjects

Six hundred sixty-two responses were recorded. After removing duplicates and incomplete responses, 421 responses were verified, and participants received gift cards. Data analyses of completed, nonduplicated surveys from participants who received a gift card are presented here. Demographic characteristics of survey participants are shown in Table 1. Surveys were completed in English (*n* = 345) and Spanish (*n* = 76) and included respondents from 15 United States, representing all 4 USDA NIFA regions. All respondents participated in ≥1 SNAP-Ed (*n* = 206) or EFNEP (*n* = 182) nutrition education class. Most respondents were the primary shopper for their household (70%), aged 26 to 57 y (72%), and female (84%). Racial and ethnic groups represented include White (46%), Hispanic or Latinx (33%), Black (17%), and American Indian/Alaskan Native, Asian, or Native Hawaiian (14%). Sixty-three percent of respondents had an annual household income of <$25,000.

**TABLE 1:** Sociodemographic characteristics of survey respondents (*n =* 421)

Characteristic	Frequency[*n* (%)]
USDA NIFA region	
Western (California, Idaho, Utah, Washington, Wyoming)	102 (24)
North Central (Iowa, Ohio, South Dakota)	155 (37)
Southern (Alabama, Oklahoma, Virginia)	75 (18)
Northeast (Maine, New Jersey, New York, Rhode Island)	89 (21)
Age (y)	
18–25	37 (9)
26–41	194 (46)
42–57	111 (26)
≥58	79 (19)
Race	
American Indian, Alaska Native, Asian, or Native Hawaiian	61 (14)
Black or African American	73 (17)
White	193 (46)
Not listed or prefer not to answer	116 (28)
Ethnicity	
Hispanic or Latinx	139 (33)
Non-Hispanic or Non-Latinx	248 (59)
Prefer not to answer	34 (8)
Gender	
Male	58 (14)
Female	355 (84)
Prefer not to answer	8 (2)
Education	
Less than high school or some high school	88 (21)
High school diploma or GED	128 (30)
Some college or 2 y degree	134 (32)
Bachelor’s degree or higher	60 (14)
Prefer not to say	11 (3)
Income	
<$15K	188 (45)
$15,000–$24,999	76 (18)
$25,000–$49,999	95 (23)
$50,000–$74,999	40 (10)
≥$75,000	19 (5)
No response	3 (>1)

Values represent the number of respondents and percentage of the total sample in each category.

Abbreviations: GED, General Education Development; NIFA, National Institute of Food and Agriculture.

### Frozen food purchases and consumption

Ninety-four percent of respondents reported purchasing FFV occasionally or all the time. Thirty-two percent reported eating frozen food (not limited to FFV) every few days or just about every day, making them heavy or core consumers, and 35% reported eating frozen foods every few weeks or less than once a month, making them light consumers. Factors influencing FFV purchases and common product types are summarized in Table 2. When asked the top 3 reasons for purchasing FFV, respondents identified long shelf life (38%), ease of preparation (36%), and convenience (34%). Health/nutrition traits that respondents looked for when purchasing FFV included health benefits (immunity, energy, etc.) (52%), nutritional value (high/low in …) (51%), and no artificial ingredients (sweeteners, preservatives, etc.) (44%). The most commonly purchased FFV were individual vegetables (61%), followed by mixed fruit (58%) and mixed vegetables (54%). Most respondents (71%) purchase FFV at big box retailers such as Wal-Mart, Target, and Fred Meyer, with fewer reporting purchasing FFV at club stores (39%) such as Costco, Sam’s Club, or BJ’s and other supermarket/grocery stores (38%) such as Kroger, HEB, Albertsons, Publix, Winco, or Safeway.

**TABLE 2:** Factors influencing frozen fruit and vegetable purchases and common product types reported by SNAP-Ed and EFNEP participants (*n =* 421)

	Frequency ofrespondents[*n* (%)]
Top 3 reasons for buying FFV	
They last much longer (shelf life)	158 (38)
Ease of preparation	152 (36)
Convenience	144 (34)
Cost	136 (32)
Time savings	112 (27)
Taste	99 (24)
Nutritional value	80 (19)
Quality	73 (17)
Variety of options	63 (15)
Product consistency	18 (4)
Health/Nutrition traits looked for when purchasing FFV	
Health benefits (immunity, energy, etc.)	220 (52)
Nutritional value (high/low in…)	216 (51)
No artificial ingredients (sweeteners, preservatives, etc.)	185 (44)
None of these	66 (16)
Other	12 (3)
Types of FFV typically purchased	
Individual vegetables, such as peas, broccoli, beans, corn, etc.	255 (61)
Mixed fruit, such as a blend of strawberries, blueberries, and raspberries	246 (58)
Mixed vegetables, such as peas and carrots or peppers and onions	227 (54)
Potato-based items, such as fries or hashbrowns	185 (44)
Individual fruits, such as peaches or mango	158 (38)
Smoothie mixes	148 (35)
Prepared vegetables, such as seasoned brussels sprouts or broccoli florets with cheese sauce	137 (33)
Fruit and yogurt bites	129 (31)
Frozen entrees with fruit and vegetables	120 (29)
Vegetables as carb alternatives, such as riced cauliflower or spiralized zucchini “noodles”	90 (21)

Values represent the number of respondents and percentage of total participants selecting each option. Respondents could select multiple responses within each section.

Abbreviations: EFNEP, Expanded Food and Nutrition Educaiton Program; FFV, frozen fruits and vegetables; SNAP-Ed, Supplemental Nutrition Assistance Program Education.

The importance of using SNAP benefits to purchase frozen foods and barriers to purchasing frozen foods in general are shown in Table 3. Fifty-nine percent of respondents said that using SNAP benefits to purchase frozen foods was very or extremely important. Stratified by consumer group, 65% of core consumers and 51% of light consumers reported that using SNAP benefits to purchase frozen foods was very or extremely important. Cost was the top barrier to purchasing frozen foods across all consumer groups (59%), followed by concerns about quality (35%). Although barriers identified for frozen foods are likely relevant to FFV as part of the broader frozen food category, Table 4 summarizes conditions that would increase the purchase of FFV specifically. Forty-seven percent would purchase more FFV if they were available in convenience or other easy in-and-out stores, and 67% would purchase more if they had more freezer space at home. Eight percent of respondents did not have a freezer of any capacity at home, and only 20% had a separate, standalone freezer.

**TABLE 3:** Importance of SNAP benefits and barriers to purchasing frozen foods by frequency of frozen food consumption (*n =* 421)

	Frequency of respondents by frozen food consumption group [*n* (%)]			
	Core (at least everyfew days) (*n =* 136)	Medium (weekly or everyother week) (*n =* 138)	Light (every few weeksor less) (*n =* 147)	All respondents
Importance of SNAP				
Extremely important	57 (42)	46 (33)	30 (20)	133 (32)
Very important	31 (23)	37 (27)	44 (30)	112 (27)
Moderately important	20 (15)	20 (15)	33 (23)	73 (17)
Slightly important	17 (12)	11 (8)	23 (16)	51 (12)
Not at all important	11 (8)	24 (17)	16 (11)	51 (12)
Barrier				
Cost	85 (63)	76 (55)	87 (59)	248 (59)
Concerns about quality	44 (32)	43 (31)	59 (40)	146 (35)
Limited availability of nutritious options	44 (32)	35 (25)	38 (26)	117 (28)
Lack of variety	38 (28)	34 (25)	36 (24)	108 (26)
Packaging size not suitable to needs	40 (29)	35 (25)	30 (20)	105 (25)
Difficulty with storage	37 (27)	30 (22)	33 (22)	100 (24)
Difficulty in what I’m looking for	29 (21)	24 (17)	29 (20)	82 (19)
Lack of cooking instructions on the packaging	25 (18)	9 (7)	28 (19)	62 (15)
Accessibility to stores selling frozen food	22 (16)	16 (12)	13 (9)	51 (12)
Other	8 (6)	9 (7)	4 (3)	21 (5)

Values represent the number of respondents and percentage of total participants within each consumption group selecting each response option. Respondents could select multiple barriers. Consumption groups were defined by self-reported frequency of frozen food consumption: core = at least every few days; medium = weekly or every other week; light = every few weeks or less.

Abbreviation: SNAP, Supplemental Nutrition Assistance Program.

**TABLE 4:** Conditions that would increase purchase of frozen fruits and vegetables by frequency of frozen food consumption (*n =* 421)

Condition	Frequency of respondents by frozen food consumption group [*n* (%)]			All respondents
	Core (at leastevery few days)(*n =* 136)	Medium (weeklyor everyother week) (*n =* 138)	Light (every fewweeks or less)(*n =* 147)	
Freezer space				
Yes, would purchase if had more freezer space at home	104 (76)	94 (69)	83 (56)	281 (67)
No, freezer space is not an issue	24 (18)	34 (25)	52 (35)	110 (26)
No, if I had more freezer space I would use it for other frozen foods	8 (6)	9 (6)	12 (8)	29 (7)
Availability in convenience stores				
Yes, I would purchase if available in convenience stores	70 (52)	64 (47)	65 (44)	199 (47)
No, these foods are already available at the convenience stores where I shop	22 (16)	31 (22)	36 (25)	89 (21)
No, I do not shop at these types of stores	33 (24)	31 (22)	22 (15)	86 (21)
No, I would not purchase more even if they were more available at these stores	11 (8)	12 (9)	23 (16)	46 (11)

Values represent the number of respondents and percentage of total participants within each consumption group selecting each response option. Consumption groups were defined by self-reported frequency of frozen food consumption: core = at least every few days; medium = weekly or every other week; light = every few weeks or less.

### FFV perceptions and use

**FIGURE 1:**
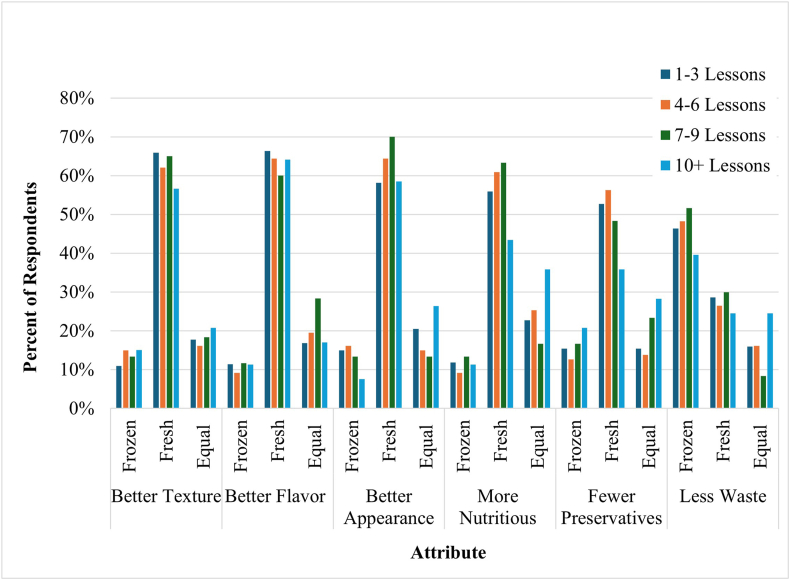
Comparison of perceived attributes of fresh and frozen fruits and vegetables by number of SNAP-Ed or EFNEP lessons completed.Participants were asked to compare attributes of produce and identify whether they perceived the attribute to apply more to fresh produce, frozen produce, or apply equally to both. Results were stratified according to the number of nutrition education lessons respondents participated in and summarized in this figure. EFNEP, Expanded Food and Nutrition Education Program; SNAP-Ed, Supplemental Nutrition Assistance Program Education.

Almost all respondents (98%) purchased fresh FV in addition to frozen. Participants were asked to compare attributes of produce and identify whether they perceived the attribute to apply more to fresh FV, FFV, or apply to both equally. As shown in Table 5, overall, respondents perceived FFV as offering less food waste (46%). However, fresh FV were perceived as having fewer preservatives (51%), better appearance (61%) and texture (64%), more nutritious (56%), and better flavor (65%). When stratified by the number of nutrition education lessons completed, as shown in Figure 1, the percentage of individuals who perceived fresh and frozen FV as equal in terms of preservatives (28%) and nutritional value (36%) was highest when individuals completed ≥10 lessons. However, χ^2^ analysis showed no significant relationships between the number of nutrition education lessons and attribute response (*P* < 0.05; Supplementary Material; Perceptions of Fresh Versus FFV by Number of Nutrition Education Lessons).

**TABLE 5:** Comparison of perceived attributes of fresh and frozen fruits and vegetables among SNAP-Ed and EFNEP participants (*n =* 421)

Attribute	Frozen [*n* (%)]	Fresh [*n* (%)]	Equal [*n* (%)]	Unsure [*n* (%)]	*P* value∗
Better texture	53 (12)	268 (64)	75 (18)	24 (6)	<0.0001
Better flavor	46 (11)	272 (65)	80 (19)	23 (5)	<0.0001
More nutritious	48 (12)	237 (56)	101 (24)	34 (8)	<0.0001
Better appearance	59 (14)	257 (61)	80 (19)	24 (6)	<0.0001
Fewer preservatives	66 (16)	213 (51)	75 (18)	65 (15)	<0.0001
Less food waste	196 (46)	117 (28)	67 (16)	41 (10)	<0.0001

Values represent the number and percentage of respondents selecting each response option when comparing fresh and frozen fruits and vegetables.

Abbreviations: EFNEP, Expanded Food and Nutrition Education Program; SNAP-Ed, Supplemental Nutrition Assistance Program Education.

∗ Χ^2^ analysis was used to test differences in frequency counts across response options.

Eighty percent of respondents reported being the one in the household who prepares meals always or most of the time. Twenty-six percent of respondents reported preparing FFV every few days to daily, 42% weekly to every other week, and 32% every few weeks to less than once a month. FFV use and preparation are summarized in Table 6. The most common reason respondents use FFV instead of fresh FV is when they need something quickly (52%), something that is easy to prepare (48%), or they have run out of fresh produce (42%). When asked whether or not specific reasons described their use of FFV, most respondents reported using FFV as a backup meal solution (84%), to help prevent food waste (82%), and to allow buying a mix of FV at once (81%). Most respondents were not concerned about freezer burn, with 68% reporting never or hardly ever throwing out FFV because they were too old or freezer-burned. The most common dishes made using FFV include beverages/smoothies (60%) and sides to a main entrée (47%). Around one-third of respondents reported using FFV in casseroles, stews/soups/chowders, breakfast foods, and desserts.

**TABLE 6:** Uses and meal applications of frozen fruits and vegetables among SNAP-Ed and EFNEP participants (*n =* 421)

Characteristic	Frequencyof respondents[*n* (%)]
Use of FFV instead of fresh	
When I need something quickly	220 (52)
When I need something easy to prepare	201 (48)
When I run out of fresh produce I use frozen	177 (42)
The specific meal/beverage/etc. that I am making	156 (37)
The type of fruit or vegetable I’m using	150 (36)
Other	13 (3)
Use of FFV specifically	
I like to have them as a backup solution	353 (84)
They help me prevent food waste	345 (82)
They allow me to buy a mix of fruits/vegetablesall at once	342 (81)
They allow me to save money over buyingfresh fruit/vegetables	319 (76)
They hold me over in between shoppingtrips when running out of fresh produce	309 (73)
They make it easier to eat more fruitsand vegetables	302 (72)
They are an easy solution for fruits/vegetablesI do not know how/want to make	288 (68)
I buy them with a specific meal/day in mind	282 (67)
Dishes made using FFV	
Beverages/smoothies	252 (60)
Sides to a main entrée	197 (47)
Casseroles	163 (39)
Soups/stews/chowders	159 (38)
Breakfast foods, such as omelets	158 (38)
Desserts	130 (31)
Pasta/rice dishes	116 (28)
Appetizers	93 (22)
Baked goods, such as pie and cobblers	93 (22)
Sauces	59 (14)
Compotes/jams/jellies	25 (6)
Other	14 (3)

Values represent the number and percentage of total participants selecting each response option. Respondents could select multiple responses.

Abbreviations: EFNEP, Expanded Food and Nutrition Education Program; FFV, frozen fruits and vegetables; SNAP-Ed, Supplemental Nutrition Assistance Program Education.

### Nutrition education and outreach

When asked about which nutrition education topics subjects would find useful and want to learn about, “how to prevent freezer burn” was the most popular (47%), followed by “how to prepare simple meals and snacks with frozen food” (43%), “how to meal plan with frozen foods” (39%), and “how to use frozen foods in recipes for a family” (36%) (Supplementary Material; Nutrition Education Topics of Interest). About one-third of respondents would also like to learn more about “how to safely store and reheat frozen foods,” “how to use frozen foods in recipes for one or two,” “how frozen foods fit into a budget,” “how to safely store bulk frozen foods,” and “how frozen foods fit into MyPlate/a healthy diet.” Fewer respondents (about one-fourth) were interested in learning “how to use frozen whole grains in meals and snacks,” “how to read a nutrition fact label or ingredient list on frozen food products,” and “how to prepare frozen seafood.”

Most respondents (60%) were somewhat or extremely likely to follow a social media platform providing education and tips about purchasing and preparing frozen foods. The most common social media sites used included Facebook (77%) and YouTube (57%), with fewer respondents using Instagram (37%), TikTok (35%), Pinterest (22%), and Twitter (8%). Booths or displays highlighting certain products were at least sometimes liked by almost all (94%) of respondents. Responses about the use of QR codes in the grocery store were mixed, with about one-third somewhat or extremely unlikely to use them and ∼49% somewhat or extremely likely to use them to find out more information about products. Most respondents had never met with a grocery store registered dietitian (85%). Respondents did report reading nutrition facts labels on frozen foods sometimes to always (82%).

## Discussion

This cross-sectional multistate study described the perceptions and behaviors related to FFV consumption among adults with low income who have participated in EFNEP or SNAP-Ed classes. Individuals participating in these educational programs are eligible to receive financial assistance through the SNAP [15]. In fact, 59% of respondents said that using SNAP benefits to purchase frozen foods, including FFV, was very or extremely important to them. When stratified by consumer base, 65% of those who eat frozen foods every few days to every day (32%, core consumers) rely on SNAP benefits for these purchases. Previous research has shown that households using SNAP were more likely to purchase frozen vegetables than households with low income not using this food assistance program [16]. When taken with previous market research that identified 25% of SNAP-eligible households as core FFV consumers [14], this underscores the importance of SNAP to provide access to these foods. Another key food assistance program to promote fruit and vegetable consumption, the Gus Shumacher Nutrition Incentive Program Produce Prescription Program, only allows for fresh, not frozen, dried, or canned FV [17]. Consideration for future incentive programs could benefit from including all forms of FV to increase impact.

Previous studies have shown mixed results when assessing the intake of specific forms of FV (i.e., fresh, frozen, and canned). Trofholz et al. [18] found that households that regularly have fresh (not canned or frozen) FV on hand are more likely to serve these foods at family meals, whereas Storey et al. [5] found that fruit and vegetable consumption is higher among those who consume frozen produce. Respondents in the current study reported purchasing both fresh and frozen FV (98%). However, they also perceived fresh FV as more nutritious and having fewer preservatives than FFV. This finding was similar to findings by researchers who conducted semistructured interviews with consumers who avoided purchasing frozen foods and identified concerns about the health of additives as well as food safety in the manufacturing process, and believed that frozen foods were less nutritious [19].

Results from the current study suggest that participants in nutrition education programs may have greater knowledge and more positive perceptions of FFV. When stratified by the number of nutrition education lessons completed, when individuals completed 10 or more lessons, the percentage of respondents who perceived fresh and frozen FV as equal in terms of nutrition and preservatives was higher. Although the number of participants completing 10 or more lessons was perhaps too low to evaluate statistical significance, and this study is cross-sectional, this observation indicates a potential for nutrition education to dispel some of the myths surrounding FFV. Future longitudinal studies are needed to assess changes in knowledge and perceptions due to participation in nutrition education classes. Work by Polacsek et al. [20] demonstrated the efficacy of combined financial incentives and nutrition education on-site in supermarkets to increase FV purchasing and consumption. Others have found that nutrition education specifically focused on the health benefits of FV in reducing the risk of chronic disease was effective in increasing intake among adults with overweight/obesity [21]. SNAP-Ed social marketing campaigns reaching mothers and children have been successful in promoting positive perceptions of FV [22,23]. These campaigns focused on overall FV promotion, without specific attention given to frozen products.

However, existing nutrition education strategies alone may not be enough to change all consumer perceptions around FFV. Dudley et al. [24] examined the implicit and explicit biases around frozen and canned FV, indicating strong implicit biases in terms of health, well-being, and preference, which may not be changed by traditional nutrition education methods. Future nutrition education strategies may include innovative ways to help change the misperceptions about both the nutritional quality and taste of FFV. Providing guidance and skills to cook and prepare FFV, which may be suited for different types of recipes than fresh FV (e.g., soups, stews, and smoothies), can help change consumer opinions about FFV’s taste and sensory profiles. Demonstrating recipes that are designed to highlight FFV, and allowing opportunities for consumers to sample these dishes, may help to change consumer perceptions.

This research has notable strengths and limitations. Regarding strengths, this study was, to the authors’ knowledge, the first to conduct frozen food consumer research on a sample of entirely SNAP-eligible individuals who have participated in federally funded nutrition education programming. The multistate design also allowed for a diverse sample of adults with low incomes from 15 states. Several other studies of this population have shown similar gender, income, and racial and ethnic distributions in their samples, suggesting that our sample is well-representative of the population participating in SNAP-Ed and EFNEP [25,26]. However, limitations in our study design also exist. Because this study was cross-sectional, we could not investigate how participants may have changed their FFV perceptions from the beginning to the end of a SNAP-Ed/EFNEP lesson series. Although the study was multistate in design, key regional differences in FFV perceptions may be missing due to some United States geographic subregions not fully represented in the sample, including the Southwest, Deep South, and the noncontiguous states and territories. However, few studies of these programs are able to achieve such a sample through prospective designs due to the time and effort associated with recruitment. Moreover, because recruitment partnerships were only developed with land-grant universities, there may be some differences in the perceptions of SNAP-Ed participants who participated in nonland-grant programs that are unaccounted for in this study.

Future studies could aim to use longitudinal designs to see how participants’ perceptions and use of FFV change over the course of a nutrition education lesson series. Moreover, novel nutrition education approaches to change perceptions and misconceptions of FFV could also be investigated, such as the development and implementation of frozen produce-specific recipe resources or a social marketing campaign. The SNAP-Ed program formally ended on 30 September, 2025, due to the passing of P.L. 119-21. Although at the time of writing, EFNEP remains as a federally funded nutrition education program, opportunities also exist for FFV nutrition education campaigns funded by trade associations or private corporations in partnership with Extension nutrition educators to test these novel strategies and expand their reach to SNAP-eligible audiences.

## Author contributions

The authors’ responsibilities were as follows – AJR, GEB: designed research; AJR: analyzed data and had primary responsibility for final content; AJR, GEB, RR: wrote the paper; and all authors: conducted research and read and approved the final manuscript.

## Data availability

Data described in the manuscript, code book, and analytic code will be made available on request by contacting the corresponding author and pending approval from the Principal Investigator.

## Funding

This study was funded by a sponsored research opportunity from the Frozen Food Foundation.

## Conflict of interest

AJR, GEM, and JKR report that financial support was provided by Frozen Food Foundation. RR declared no known competing financial interests or personal relationships that could have appeared to influence the work reported in this paper.
