# Supplementary material for: Frozen Fruit and Vegetable Perceptions and Usage among a Multistate Sample of Supplemental Nutrition Assistance Program Education and Expanded Food and Nutrition Education Program Participants
Source: Curr Dev Nutr. 2026 Jan 17;10(2):107640. doi: 10.1016/j.cdnut.2026.107640 (PMC12915165; doi:10.1016/j.cdnut.2026.107640)
Supplement: multimedia component 1 [file mmc1.pdf]

Frozen Food Qualtrics Survey Questions

In which state or territory do you currently reside?

▼ Alabama ... I do not reside in the United States (53)

Please provide the code given to you by your SNAP-Ed or EFNEP educator.

____

Throughout the survey, **frozen foods** are items you find in the frozen aisles of the grocery store, including ice cream, pizzas, prepared meals or entrees, fries or other potato products, breakfast items, fruits, vegetables, and meat/seafood. Not included are bagged ice and items you froze yourself. How often does your household eat frozen food by this definition?

- Never
- Less than once a month
- Every few weeks
- Weekly
- Every few days
- Just about every day

How many SNAP-Ed nutrition lessons have you taken?

- None
- 1-3
- 4-6
- 7-9
- 10 or more

How many EFNEP nutrition lessons have you taken?

- ◯ None
- ◯ 1-3
- ◯ 4-6
- ◯ 7-9
- ◯ 10 or more

How important are the following to you when choosing foods to purchase at the grocery store?

	Not at all important	Slightly important	Moderately important	Very important	Extremely important	Not applicable
Aligns with dietary preferences (example: vegetarian, vegan)	◯	◯	◯	◯	◯	◯
Allergen free	◯	◯	◯	◯	◯	◯
Cost	◯	◯	◯	◯	◯	◯
Nutritional value	◯	◯	◯	◯	◯	◯
Taste	◯	◯	◯	◯	◯	◯
Long shelf-life	◯	◯	◯	◯	◯	◯
Easy to prepare	◯	◯	◯	◯	◯	◯
In-season	◯	◯	◯	◯	◯	◯
Versatile (ingredient can be used in a variety of recipes)	◯	◯	◯	◯	◯	◯

Few preservatives	○	○	○	○	○	○
Organic	○	○	○	○	○	○
Variety	○	○	○	○	○	○
WIC approved	○	○	○	○	○	○
Brand name	○	○	○	○	○	○
Packaging	○	○	○	○	○	○
Aligns with cultural preferences	○	○	○	○	○	○

How much do each of the following influence your **frozen food** purchases? ***Frozen foods*** *are items you find in the frozen aisles of the grocery store, including ice cream, pizzas, prepared meals or entrees, fries or other potato products, breakfast items, fruits, vegetables, and meat/seafood. Not included are bagged ice and items you froze yourself.*

	None at all	A little	A moderate amount	A lot	A great deal
Convenience/ ease of preparation	○	○	○	○	○
On sale/ coupon	○	○	○	○	○
Attractive packaging	○	○	○	○	○
Nutrition facts panel and ingredient list	○	○	○	○	○
Familiar foods	○	○	○	○	○
On my grocery list	○	○	○	○	○

Recommendations from friends/ family					
Cost					
Quality					
Microwave-ready (steam) packaging					
Brand reputation					
Availability					
Pack size/ amount options					
Shelf life					
Environmentally friendly packaging					
Re-sealable (open/close) packaging (17)					
Recipes and meal ideas on the package (18)					

Which production-related claims matter to you when buying **frozen foods**? Please check all that apply. ***Frozen foods*** *are items you find in the frozen aisles of the grocery store, including ice cream, pizzas, prepared meals or entrees, fries or other potato products, breakfast items, fruits, vegetables, and meat/seafood. Not included are bagged ice and items you froze yourself.*

- ☐ Grown in the USA
- ☐ Fresh frozen
- ☐ No artificial ingredients (colors, flavors, sweeteners, preservatives, etc.)
- ☐ Organic
- ☐ Kosher/Halal
- ☐ Vegetarian/Vegan
- ☐ Non-GMO
- ☐ Natural
- ☐ Fair trade
- ☐ Low carb/Paleo or Keto diet friendly
- ☐ Minimally processed
- ☐ Seasonally harvested/picked
- ☐ None of these
- ☐ Other (please specify)

____

How important is it to you to be able to use SNAP (food stamps) or other benefits to purchase **frozen foods**? ***Frozen foods*** *are items you find in the frozen aisles of the grocery store, including ice cream, pizzas, prepared meals or entrees, fries or other potato products, breakfast items, fruits, vegetables, and meat/seafood. Not included are bagged ice and items you froze yourself.*

○ Not at all important

○ Slightly important

○ Moderately important

○ Very important

○ Extremely important

What barriers or challenges, if any, do you face when purchasing **frozen foods**? Select all that apply. ***Frozen foods*** *are items you find in the frozen aisles of the grocery store, including ice cream, pizzas, prepared meals or entrees, fries or other potato products, breakfast items, fruits, vegetables, and meat/seafood. Not included are bagged ice and items you froze yourself.*

☐ Cost

☐ Lack of variety

☐ Concerns about quality

☐ Accessibility to stores selling frozen food

☐ Limited availability of nutritious options

☐ Difficulty in what I'm looking for

☐ Lack of cooking instructions on the packaging

☐ Difficulty with storage

☐ Package size not suitable to needs

☐ Other (please specify)

____

Do you have any other comments or suggestions related to the barriers to purchasing **frozen foods** using SNAP dollars or other benefits? ***Frozen foods*** *are items you find in the frozen aisles of the grocery store, including ice cream, pizzas, prepared meals or entrees, fries or other potato products, breakfast items, fruits, vegetables, and meat/seafood. Not included are bagged ice and items you froze yourself.*

____

Do you have any suggestions for how SNAP or other benefit programs can better support the purchase of healthy **frozen food** options? ***Frozen foods*** *are items you find in the frozen aisles of the grocery store, including ice cream, pizzas, prepared meals or entrees, fries or other potato products, breakfast items, fruits, vegetables, and meat/seafood. Not included are bagged ice and items you froze yourself.*

____

Do you purchase **frozen fruits and vegetables**? ***Frozen fruits and vegetables*** *include individual vegetables, mixed vegetables, potato-based items, mixed fruit, individual fruit, prepared vegetables, spiralized/riced vegetables, smoothie mixes, and fruit & yogurt bites.*

○ No, never

○ Yes, occasionally

○ Yes, all the time

What types of **frozen fruits and/or vegetables** do you typically purchase? Please check all that apply.

- ☐ Individual vegetables, such as peas, broccoli, beans, corn, etc.
- ☐ Mixed vegetables, such as peas & carrots or peppers & onions
- ☐ Prepared vegetables, such as seasoned Brussels sprouts or broccoli florets with cheese sauce
- ☐ Potato-based items, such as fries or hashbrowns
- ☐ Vegetables as carb alternatives, such as riced cauliflower or spiralized zucchini "noodles"
- ☐ Individual fruits, such as peaches or mango
- ☐ Mixed fruit, such as a blend of strawberries, blueberries, and raspberries
- ☐ Fruit and yogurt bites
- ☐ Frozen entrees with fruit and vegetables
- ☐ Smoothie mixes

Where do you purchase **frozen fruit and/or vegetables**? Please check all that apply. ***Frozen fruits and vegetables*** *include individual vegetables, mixed vegetables, potato-based items, mixed fruit, individual fruit, prepared vegetables, spiralized/riced vegetables, smoothie mixes, and fruit & yogurt bites.*

- ☐ Kroger, HEB, Albertsons, Publix, Winco, Safeway, or other supermarket/ grocery store
- ☐ Walmart, Target, Fred Meyer, or other big box retailer
- ☐ Costco, Sam's Club, BJ's, or other club store
- ☐ Aldi, Lidl, Dollar General or other value-focused stores
- ☐ Whole Foods, Sprouts or other specialty/organic stores
- ☐ CVS, Walgreens, Rite Aid or other drug stores
- ☐ 7-Eleven, Casey’s, Wawa, or other convenience stores
- ☐ Amazon, SmoothieBox, Daily Harvest, or other online-only companies
- ☐ Other (please specify)

____

What are your three main reasons for buying **frozen fruits and/or vegetables**? Pick three only. ***Frozen fruits and vegetables*** *include individual vegetables, mixed vegetables, potato-based items, mixed fruit, individual fruit, prepared vegetables, spiralized/riced vegetables, smoothie mixes, and fruit & yogurt bites.*

- ☐ Quality
- ☐ They last much longer (shelf life)
- ☐ Convenience
- ☐ Cost
- ☐ Ease of preparation
- ☐ Time savings
- ☐ Taste
- ☐ Variety of options
- ☐ Nutritional value
- ☐ Product consistency

Which of these health/nutrition traits do you look for when buying **frozen fruits and/or vegetables**? Please check all that apply. ***Frozen fruits and vegetables*** *include individual vegetables, mixed vegetables, potato-based items, mixed fruit, individual fruit, prepared vegetables, spiralized/riced vegetables, smoothie mixes, and fruit & yogurt bites.*

- ☐ Nutritional value (high/low in ....)
- ☐ No artificial ingredients (sweeteners, preservatives, etc.)
- ☐ Health benefits (immunity, energy, etc.)
- ☐ None of these
- ☐ Other (please specify)

____

Thinking about purchasing frozen fruits and vegetables in the next few months, do you think you will be buying...?

- ○ A lot less frozen fruit/vegetables
- ○ Somewhat less
- ○ About the same
- ○ Somewhat more
- ○ A lot more frozen fruit/vegetables

Would you purchase more **frozen fruits and/or vegetables** if you had the freezer space?

- ○ Yes
- ○ No, freezer space is not an issue
- ○ No, if I had more freezer space I would use it for other frozen foods

Would you purchase more **frozen fruits and/or vegetables** if they were available in convenience stores or other easy in and out stores?

- ◯ Yes
- ◯ No, these foods are already available at the convenience stores where I shop
- ◯ No, I do not shop at these types of stores
- ◯ No, I would not purchase more even if they were more available at these stores

Do you purchase **fresh fruits and vegetables** as well?

- ◯ Yes
- ◯ No

Choose whether the following food attributes apply more to frozen or fresh fruits and vegetables.

	Frozen Fruits and Vegetables	Fresh Fruits and Vegetables	Frozen and Fresh are Equal	Unsure
Better texture	◯	◯	◯	◯
Better flavor	◯	◯	◯	◯
More nutritious	◯	◯	◯	◯
Better appearance	◯	◯	◯	◯
Fewer preservatives	◯	◯	◯	◯
Less food waste	◯	◯	◯	◯

What determines when you use **frozen fruits and vegetables** instead of **fresh** ones? Please check all that apply.

- ☐ When I need something quickly
- ☐ When I run out of fresh produce I use frozen
- ☐ When I need something easy to prepare
- ☐ The specific meal/beverage/etc. that I am making
- ☐ The type of fruit or vegetable I'm using
- ☐ Other (please specify)

____

Do the following describe your use of **frozen fruits and vegetables**, specifically? ***Frozen fruits and vegetables*** *include individual vegetables, mixed vegetables, potato-based items, mixed fruit, individual fruit, prepared vegetables, spiralized/riced vegetables, smoothie mixes, and fruit & yogurt bites.*

	Yes	No
I buy them with a specific meal/day in mind	○	○
I like to have them as a backup solution	○	○
They hold me over in between shopping trips when running out of fresh produce	○	○
They make it easier to eat more fruits and vegetables	○	○
They help me prevent food waste	○	○
They allow me to save money over buying fresh fruit/vegetables	○	○
They allow me to buy a mix of fruits/vegetables all at once	○	○
They are an easy solution for fruits/vegetables I do not know how/want to make	○	○

How often are you the person who prepares meals in your household?

○ Always

○ Most of the time

○ About half the time

○ Sometimes

○ Never

How often do you prepare **frozen fruits and/or vegetables**? ***Frozen fruits and vegetables*** *include individual vegetables, mixed vegetables, potato-based items, mixed fruit, individual fruit, prepared vegetables, spiralized/riced vegetables, smoothie mixes, and fruit & yogurt bites.*

- ○ Less than once a month
- ○ Every few weeks
- ○ Every other week
- ○ Weekly
- ○ Every few days
- ○ Daily

What do you make using **frozen fruits and/or vegetables**? (select all that apply) ***Frozen fruits and vegetables*** *include individual vegetables, mixed vegetables, potato-based items, mixed fruit, individual fruit, prepared vegetables, spiralized/riced vegetables, smoothie mixes, and fruit & yogurt bites.*

- ☐ Appetizers
- ☐ Baked goods, such as pie and cobblers
- ☐ Beverages/smoothies
- ☐ Breakfast foods, such as omelets
- ☐ Casseroles
- ☐ Compotes/jams/jellies
- ☐ Desserts
- ☐ Pasta/rice dishes
- ☐ Sauces
- ☐ Sides to a main entree
- ☐ Soups/stews/chowders
- ☐ Other (please specify)

____

Based on the text below, what would be your favorite vegetable? It is important to eat 2 to 3 servings of vegetables each day. We want to make sure you are paying attention. So, please choose "cauliflower" as the answer to this question.

- ○ Carrots
- ○ Broccoli
- ○ Lettuce
- ○ Cauliflower
- ○ Eggplant

How often do you throw **frozen fruits and/or vegetables** away because they are too old or freezer burnt?

- ○ Never
- ○ Hardly ever
- ○ Sometimes
- ○ Frequently

What topics would you find useful and want to learn about? (select all that apply)

- ☐ How to read a nutrition facts label or ingredient list on frozen food products
- ☐ How to prepare simple meals and snacks with frozen foods
- ☐ How to safely store and reheat frozen foods
- ☐ How frozen foods fit into MyPlate/ a healthy diet (18)
- ☐ How to prepare frozen seafood
- ☐ How to use frozen foods in recipes for one or two
- ☐ How to use frozen foods in recipes for a family
- ☐ How to safely store bulk frozen foods
- ☐ How to meal plan with frozen foods
- ☐ How to use frozen whole grains in meals and snacks
- ☐ How frozen foods fit into a budget
- ☐ How to prevent freezer burn (19)

What other topics related to frozen foods would you like to learn about?

____

If you saw a display in the grocery store, what catches your attention to stop? (select all that apply)

- ☐ Hand outs
- ☐ Free samples
- ☐ Free education from a professional
- ☐ Coupons
- ☐ Other (please specify)

____

- ☐ Nothing would make me stop at a display

What social media sites do you currently use? (select all that apply)

- ☐ YouTube
- ☐ Facebook
- ☐ Instagram
- ☐ Twitter
- ☐ TikTok
- ☐ Blog sites
- ☐ Pinterest
- ☐ Other (please specify)

____

How likely would you be to follow a social media platform, such as Instagram or Facebook, that gives tips on navigating the frozen food aisles of the grocery store, preparing recipes with frozen foods, budgeting and meal planning with frozen foods, and food safety of frozen foods?

- ◯ Extremely unlikely
- ◯ Somewhat unlikely
- ◯ Neither likely nor unlikely
- ◯ Somewhat likely
- ◯ Extremely likely

Do you like booths or displays in the grocery store that highlight a certain product?

- ◯ Yes
- ◯ Sometimes
- ◯ No

How likely would you be to use a QR code on a grocery store display to link to more information online (such as a recipe video or nutrition information)?

- ◯ Extremely unlikely
- ◯ Somewhat unlikely
- ◯ Neither likely nor unlikely
- ◯ Somewhat likely
- ◯ Extremely likely

How often do you read the labels on frozen food products?

- ◯ Always
- ◯ Often
- ◯ Sometimes
- ◯ Rarely
- ◯ Never

Have you or a member of your household met with a Registered Dietitian at a grocery store or supermarket?

- ◯ Yes
- ◯ No

Which best describes your role for groceries for your household?

- ◯ I do most or all of the grocery shopping
- ◯ Someone else does most or all the grocery shopping
- ◯ I share grocery shopping responsibilities equally with other members of my household

Who do you shop with most often?

- ◯ Alone
- ◯ Another adult
- ◯ Children
- ◯ Whole family
- ◯ Other (please specify) ____

How do you travel to get groceries?

- ◯ Drive own car
- ◯ Ride with a family member or friend in their car
- ◯ Bus
- ◯ Taxi, Uber, Lyft or other ride share
- ◯ Walk
- ◯ Bike
- ◯ Other (please specify) ____

How many miles do you travel to your nearest grocery store?

- ◯ 1-5 miles
- ◯ 6-15 miles
- ◯ 16-25 miles
- ◯ 26-40 miles
- ◯ 41-60 miles
- ◯ More than 60 miles

Is your nearest grocery store your preferred grocery store?

- ◯ Yes
- ◯ No

How many miles to your preferred grocery store?

- ○ 1-5 miles
- ○ 6-15 miles
- ○ 16-25 miles
- ○ 26-40 miles
- ○ 41-60 miles
- ○ More than 60 miles

Why do you prefer the grocery store located further away?

- ☐ The grocery store located further away is more affordable
- ☐ The grocery store located further away has my desired and preferred grocery items
- ☐ Other (please specify) ____________________

How often do you go grocery shopping?

- ○ 5-7 times a week
- ○ 3-4 times a week
- ○ 1-2 times a week
- ○ Every other week
- ○ Other (please specify) ____________________

How would you best describe your shopping habits?

- ◯ Lots of quick fill-in trips
- ◯ Few bulk buy trips
- ◯ Combination of bulk and fill-in trips
- ◯ 1 item shopping trips
- ◯ Other (please specify) ____

How often did you order online for grocery pick-up or delivery from a local store in the past year?

- ◯ Always
- ◯ Most of the time
- ◯ About half the time
- ◯ Sometimes
- ◯ Never

What is your freezer capacity at home? If you have more than one, please check all the types of freezers you have.

- ☐ No freezer/freezer compartment at all
- ☐ A small fridge with a small (shoebox size) freezer compartment
- ☐ A large fridge with a freezer drawer or compartment
- ☐ Multiple large fridges with freezer drawers/compartments
- ☐ Additional standalone freezer
- ☐ Multiple standalone freezers
- ☐ Other (please specify)

____

Based on the text below, what would be your favorite brand of soda/pop? It is important to limit the amount of soda or pop we drink, as it is very high in sugar. We want to make sure you are paying attention, so please choose "Dr. Pepper" as the answer to this question.

- ○ Coca-Cola
- ○ Fanta
- ○ Dr. Pepper
- ○ Pepsi
- ○ Mountain Dew

These next questions ask about you and your family. Remember all answers are confidential.

Which of the following programs did you or your family participate in over the past year? (Check all that apply)

- ☐ Free or reduced school lunch or breakfast
- ☐ Food Distribution Program on Indian Reservations (FDPIR)
- ☐ Head Start
- ☐ SNAP (EBT, food stamps)
- ☐ SNAP-Ed
- ☐ Temporary Assistance for Needy Families (TANF)
- ☐ WIC
- ☐ Commodity programs
- ☐ Used a food bank, food pantry, or soup kitchen
- ☐ Other (please specify)

____

- ☐ None of the above

Including yourself, how many people are in your household? (Household is defined as those currently living within your home, including family and non-family members, at least half the time).

____

Including yourself, select the following age groups of people who currently live in your household, at least half the time. (select all that apply)

- ☐ Adults 65 and older
- ☐ Adults 50-64
- ☐ Adults 18-49
- ☐ Children 5-17
- ☐ Children under 5

What is your age?

- ○ 18-25 years
- ○ 26-31 years
- ○ 32-41 years
- ○ 42-57 years
- ○ 58-67 years
- ○ 68-75 years
- ○ 76 years or older

What is your race (please select all that apply)

- ☐ American Indian or Alaska Native
- ☐ Asian
- ☐ Black or African American
- ☐ Native Hawaiian
- ☐ White
- ☐ Not listed (please specify)

____

- ☐ Prefer not to answer

What is your ethnicity

- ○ Hispanic/Latinx
- ○ Non-Hispanic/Non-Latinx
- ○ Prefer not to answer

What is your gender?

- ○ Male
- ○ Female
- ○ Non-binary / third gender
- ○ Prefer not to say
- ○ Not listed (please specify)

What is the highest degree or level of school you have completed?

- ○ Less than high school
- ○ Some high school (no diploma)
- ○ High school diploma or GED
- ○ Some college, but no degree
- ○ 2 year degree (Associate's degree)
- ○ 4 year degree (Bachelor's degree)
- ○ Master's degree
- ○ Doctorate or professional degree (for example PhD, MD)
- ○ Prefer not to say

What was your total household income before taxes in the past year?

- ◯ Less than $15,000
- ◯ $15,000 - $24,999
- ◯ $25,000 - $34,999
- ◯ $35,000 - $49,999
- ◯ $50,000 - $74,999
- ◯ $75,000 - $99,999
- ◯ $100,000 - $149,999
- ◯ $150,000 - $199,999
- ◯ $200,000 and above

Perceptions of Fresh Versus Frozen Fruits and Vegetables by Number of Nutrition Education Lessons

	**1-3 Lessons (*n*=220)**				**4-6 Lessons (*n*=88)**				**7-9 Lessons (*n*=60)**				**10+ Lessons (*n*=53)**				
**Attribute**	Frozen	Fresh	Equal	Unsure	Frozen	Fresh	Equal	Unsure	Frozen	Fresh	Equal	Unsure	Frozen	Fresh	Equal	Unsure	p-value
Better Texture	24 (11%)	145 (66%)	39 (18%)	12 (5%)	13 (15%)	54 (62%)	14 (16%)	6 (7%)	8 (13%)	39 (65%)	11 (18%)	2 (3%)	8 (15%)	30 (57%)	11 (21%)	4 (8%)	0.95
Better Flavor	25 (11%)	146 (66%)	37 (17%)	12 (5%)	8 (9%)	56 (64%)	17 (19%)	7 (8%)	7 (12%)	36 (60%)	17 (28%)	0 (0%)	6 (11%)	34 (64%)	9 (17%)	4 (8%)	0.46
More Nutritious	26 (12%)	123 (56%)	50 (23%)	21 (10%)	8 (9%)	53 (61%)	22 (25%)	4 (5%)	8 (13%)	38 (63%)	10 (17%)	4 (7%)	6 (11%)	23 (43%)	19 (36%)	5 (9%)	0.37
Better Appearance	33 (15%)	128 (58%)	45 (20%)	14 (6%)	14 (16%)	56 (64%)	13 (15%)	4 (5%)	8 (13%)	42 (70%)	8 (13%)	2 (3%)	4 (8%)	31 (58%)	14 (26%)	4 (8%)	0.52
Fewer Preservatives	34 (15%)	116 (53%)	34 (15%)	36 (16%)	11 (13%)	49 (56%)	12 (14%)	15 (17%)	10 (17%)	29 (49%)	14 (24%)	6 (10%)	11 (21%)	19 (36%)	15 (28%)	8 (15%)	0.23
Less Food Waste	102 (46%)	63 (29%)	35 (16%)	20 (9%)	42 (48%)	23 (26%)	14 (16%)	9 (10%)	31 (52%)	18 (30%)	5 (8%)	6 (10%)	21 (40%)	13 (25%)	13 (25%)	6 (11%)	0.71

Participants were asked to compare attributes of produce and identify whether they perceived the attribute to apply more to fresh produce, frozen produce, or apply equally to both. Results were stratified according to the number of nutrition education lessons respondents participated in and frequencies recorded in this table. Chi-square analysis was used to assess relationships between number of nutrition education lessons and attribute responses.

Nutrition Education Topics of Interest

Topic	Response Frequency n (%)
How to prevent freezer burn	196 (47%)
How to prepare simple meals and snacks with frozen foods	182 (43%)
How to plan meals with frozen foods	165 (39%)
How to use frozen foods in recipes for a family	152 (36%)
How to safely store and reheat frozen foods	142 (34%)
How to use frozen foods in recipes for one or two	131 (31%)
How frozen foods fit into a budget	124 (29%)
How to safely store bulk frozen foods	122 (29%)
How frozen foods fit into MyPlate/ a healthy diet	120 (29%)
How to use frozen whole grains in meals and snacks	110 (26%)
How to read a nutrition facts label or ingredient list on frozen food products	109 (26%)
How to prepare frozen seafood	106 (25%)

N=421; participants could select multiple
